# Improving Resident Self-Efficacy in Tracheostomy Management Using a Novel Curriculum

**DOI:** 10.15766/mep_2374-8265.11010

**Published:** 2020-11-03

**Authors:** J. Benjamin, K. Roy, G. Paul, S. Kumar, E. Charles, E. Miller, H. Narsi-Prasla, J. D. Mahan, S. Thammasitboon

**Affiliations:** 1 Assistant Professor, Department of Pediatrics, Baylor College of Medicine and Texas Children's Hospital; 2 Medical Director-TICU, Baylor College of Medicine and Texas Children's Hospital; Assistant Professor of Pediatrics, Department of Pediatric ICU, Texas Children's Hospital and Baylor College of Medicine; 3 Assistant Professor, Department of Pulmonology, Nationwide Children's Hospital and the Ohio State University College of Medicine; 4 Instructor, Department of Pediatrics, Baylor College of Medicine and Texas Children's Hospital; 5 Nurse Practitioner, Department of Pediatrics, Baylor College of Medicine and Texas Children's Hospital; 6 Nurse Practitioner, Department of Otolaryngology, Baylor College of Medicine and Texas Children's Hospital; 7 Associate Director, Center for Faculty Advancement, Mentoring and Engagement (FAME), the Ohio State University College of Medicine; Professor, Department of Pediatrics, Nationwide Children's Hospital and the Ohio State University College of Medicine; Program Director, Pediatric Nephrology Fellowship Programs, Nationwide Children's Hospital and the Ohio State University College of Medicine; 8 Associate Professor and Director, Center for Research, Innovation and Scholarship (CRIS) in Medical Education, Baylor College of Medicine and Texas Children's Hospital

**Keywords:** Tracheostomy Management, Simulation, Self-Efficacy, Airway Management, Airway Emergency, Critical Care Medicine, Otolaryngology, Pediatrics, Primary Care, Clinical Teaching/Bedside Teaching

## Abstract

**Introduction:**

Patients receiving pediatric tracheostomy have significant risk for mortality due to compromised airway. Timely management of airway emergencies in children with tracheostomies is an important clinical skill for pediatricians. We developed this curriculum to improve residents’ self-efficacy with tracheostomy management.

**Methods:**

We collected baseline data on 67 residents from two hospitals while creating a blended curriculum with video-based instruction on routine tracheostomy change and team management of tracheostomy emergency. Forty residents enrolled in the curriculum. During an ICU rotation, they received face-to-face instruction on routine tracheostomy change in small groups, followed by assessment of managing a tracheostomy emergency during a simulation. A video completed prior to the simulation took 9 minutes, the routine tracheostomy change didactic session took 15 minutes, and the simulation instruction was completed in 10–15 minutes. We collected feedback on the effectiveness of the curriculum from the participants.

**Results:**

All 107 residents from the baseline and intervention groups completed the self-efficacy survey. The intervention group had significantly higher changes in scores across all self-efficacy domains than the baseline group. On the curriculum feedback survey, residents rated the curriculum very highly, between 4.4 and 4.8 on a 5-point Likert scale.

**Discussion:**

Our blended curriculum increased learners’ self-efficacy and promoted learner competence in tracheostomy management. Residents scored more than 80% across all aspects of simulation assessment and reported higher self-efficacy scores following our curricular intervention.

## Educational Objectives

By the end of the session, learners should be able to:

1. Demonstrate the steps involved in providing routine tracheostomy care.
2. Provide appropriate suctioning through tracheostomy.
3. List the four steps to follow in a tracheostomy emergency.
4. Execute cognitive, technical, and behavioral components of managing tracheostomy during simulation.

## Introduction

Patients with tracheostomies have a significant risk for mortality due to loss of airway, prompting the need for efficient and timely management.^1,2^ With an increasing number of tracheostomies being performed for chronic home ventilation,^3^ numerous studies have looked at mortality rates related to tracheostomies associated with accidental decannulations, cannula obstruction, and misplacement.^4–7^ Many studies show a lack of knowledge in pediatric providers, including emergency physicians and primary care providers, regarding managing tracheostomy-related emergencies, and yet, formal education addressing this educational gap is limited.^4,8–10^ Additional studies of health care professionals report their lack of confidence and feeling underprepared to perform a tracheostomy change.^8^ Previously published curricula have addressed tracheostomy emergencies without specifically considering the need for hands-on skills and self-efficacy (SE)^11^ or using simulation to develop tracheostomy management skills.^12^ Our curriculum is unique in that it has been developed with the specific aim of improving residents’ SE by teaching hands-on skills for routine care of tracheostomy and using a stepwise approach for managing tracheostomy emergencies.

Using the SE theory posited by Bandura,^13^ we created a survey tool addressing different aspects of SE with management of tracheostomies. SE is defined as a person's confidence in his or her own abilities and how that confidence can influence the events in his or her life. This SE survey investigated knowledge, identifying emergencies, performance, and coping skills through a self-reported assessment at the beginning and end of every clinical rotation during which residents cared for patients with tracheostomies. The aim was to identify gaps in residents’ curriculum and to simplify management of a patient with tracheostomy.

**Figure 1.**
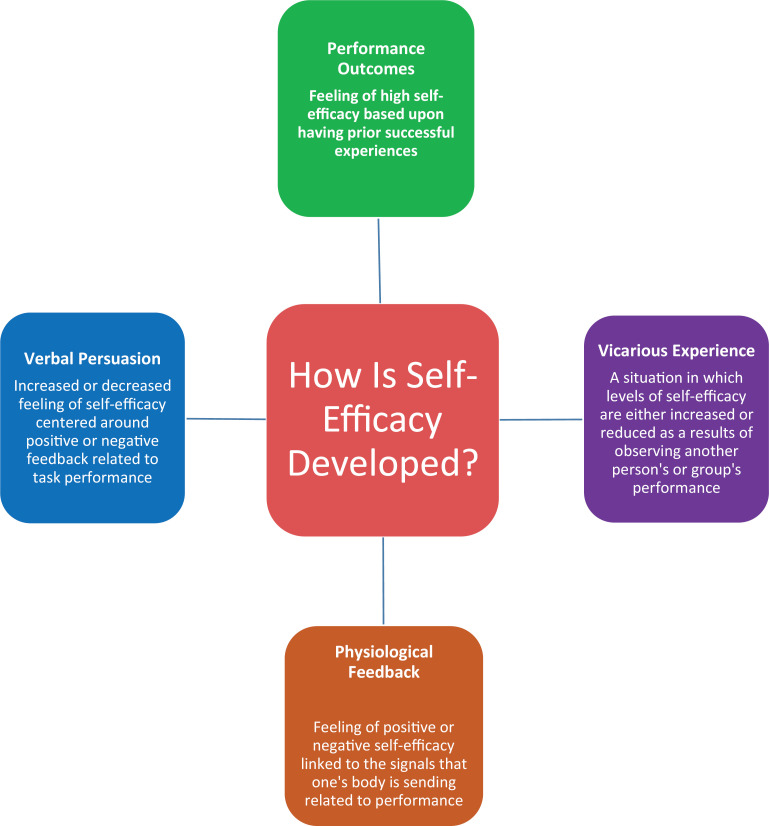
The conceptual framework of self-efficacy principles as outlined by Bandura.

We applied the different principles of SE (Figure 1) when designing this curriculum, which comprised multiple components, and used SE as the guiding principle as follows:

• Physiological feedback (feeling of positive SE linked to the signals one's body is sending related to task performance): We ensured a positive supportive learning environment by providing cues to improve physiological feedback to enhance SE.
• Verbal persuasion (increased SE based on positive or negative feedback related to task performance): We intentionally used verbal persuasion through constructive feedback during debriefing of simulation sessions.
• Vicarious experiences (increased SE as a result of observing peer- or instructor-led instruction and performance): We allowed opportunities for learners themselves to perform and demonstrate tracheostomy change during group-based exercises.
• Performance outcome (increased SE based on prior successful performances): We highlighted successful performance to individual learners throughout the learning experience.

Simulation with deliberate practice, expert-led instruction, and debriefing has been useful in improving providers’ confidence, with retention of resuscitation skills, enhanced outcomes of knowledge and behavioral skills, and a desire for improved hands-on training.^14–20^ Applying these principles, residents received expert-led instruction with a video-based asynchronous curriculum, followed by hands-on, simulation-based teaching allowing for deliberate practice and constructive feedback. The reiteration of content covered in the video module allowed for developing cognitive, technical, and behavioral skills that were assessed during simulation. Our curriculum is unique in specifically addressing a gap in training by promoting pediatric residents’ SE with management of tracheostomies. We used an SE-assessment survey to validate this context and implemented hands-on skills training of airway management to address the gap in the curriculum. In addition, we sought to complement the established procedures with the unique aspect of developing SE in this very vital skill.

## Methods

There was no prerequisite to complete this tracheostomy curriculum, but its relevance in training addressed skills pertaining to a wide range of learners, from medical student to physicians and residents alike. We collected pre- and postrotation surveys during the first year of collecting baseline data, during which time we developed our educational intervention. This educational resource was a blended curriculum with video-based instruction of routine tracheostomy change and team management of tracheostomy emergency. During the rotation, residents received face-to-face instructions on routine tracheostomy change in small groups of four or five learners, followed by assessment of managing a tracheostomy emergency during a simulation. The video completed prior to simulation took 9 minutes, the routine tracheostomy change didactic session took 15 minutes, and the emergency tracheostomy simulation instruction was completed in 10–15 minutes by each learner.

### Implementation of the Module

The SE survey (Appendix A) was a 14-item questionnaire developed by Pruitt et al. It covered four domains of SE: knowledge, identifying emergencies, performance, and coping. These questionnaires could be administered as online surveys or in paper form during face-to-face interactions. We administered the questionnaires to the residents at the beginning and completion of their rotations. We requested that the residents answer the 14-item presurvey prior to starting their ICU rotation and prior to watching the video module, then answer the same SE survey following their rotation. Learners included pediatric resident trainees from all years, medical students, and anesthesia and medicine/pediatrics residents, rotating through the transitional ICU (TICU) and complex care department.

**Figure 2.**
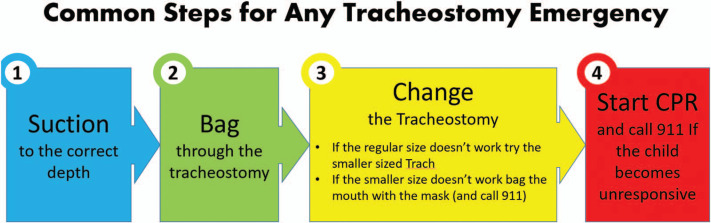
Steps to follow for a tracheostomy emergency.

The video module (Appendix B) demonstrated different aspects of routine care of a tracheostomy, including indications and supplies needed for tracheostomy change and types of tracheostomy tubes, as well as providing step-by-step instruction for managing tracheostomy emergencies. The video had two components: routine change and suctioning of a patient with a tracheostomy and a simulated scenario of a tracheostomy emergency. The module highlighted a simplified four-step approach (Figure 2) to managing a tracheostomy emergency. To prepare residents for managing a patient with a tracheostomy and allow them to apply their learned skills during the rotation, we gave them instructions to access the module prior to starting their TICU rotation.

The knowledge assessment (Appendix C) was a 10-item cognitive test, administered online to assess whether residents grasped the content covered in the video module. The assessment was a multiple-choice questionnaire, and residents received immediate feedback with explanations to their responses. We created this assessment and piloted it with a group of faculty, residents, and chief residents, who provided feedback. We incorporated their input prior to implementing the questionnaire, which we administered on REDCap, allowing for data retrieval. We trained faculty instructors to conduct the simulation instruction in a standardized fashion and showed them how to grade performance.

### Personnel and Equipment

We held simulation instruction (Appendix D) and assessment (Appendix E) in a simulation lab that had a high-fidelity manikin with the ability to control observation settings such as heart rate, saturations, and respiratory rate. To implement the simulation instruction (Appendix D), faculty instructors had to be able to operate the computer controls and to provide care only as instructed by the learner during the simulation scenario. We advised residents to think out loud, and instructors helped with redirecting in case any resident diverged from the expected responses. Instructors demonstrated the four steps (Figure 2) to follow in any tracheostomy-related emergency. We limited the number of instructors to four to reduce interrater variability. The instructors were familiar with routine tracheostomy care and were well versed in the stepwise approach to managing tracheostomy emergencies.

We performed the simulation instruction (Appendix D) with a small group of five or six residents at a time, with one faculty instructor demonstrating tracheostomy change, after which each resident assisted in the two-person technique of a routine tracheotomy change. Instructors encouraged residents to assist each other in the two-person technique in order to give them opportunities for deliberate practice. After allowing the residents to use hands-on practice on a mannikin, faculty instructors asked residents to demonstrate routine change, which the instructors assessed by direct observation. Faculty instructors also used direct observation for simulation assessment (Appendix E) of routine care as part of group hands-on education. Following this, faculty instructors evaluated each resident individually using the simulation scoring sheet for performance on a tracheostomy emergency scenario progressing onto decompensation.

We performed the assessment (Appendix E) in a dedicated simulation lab using an integrated clinical simulator, which was instructor driven on a high-fidelity Gaumard manikin. Instructors were familiar with how to provide prompts if needed (e.g., providing cues about abnormal vital signs and response to treatment) with closed-loop communication and feedback cues during the scenario. Following simulation, residents received immediate constructive feedback with debriefing, allowing for verbal persuasion. Residents provided feedback on the quality of the curriculum in a 10-item survey (Appendix F) along with narrative comments. The survey captured four domains: process, content, learning environment, and outcome. Residents also completed the SE survey at the end of the rotation. The SE survey results reflected overall impact of the entire blended learning.

### Evaluation

We collected the pre- and postrotation surveys during the first year of collecting baseline data, during which time we developed our educational resource. After obtaining IRB approval, we collected baseline data on residents’ SE for 12 months and reported on the first 6 months of intervention with simulation from June 2017 to December 2018. We encouraged residents to complete the SE survey, video module, and knowledge assessment prior to starting their clinical rotations in the TICU, the in-patient unit for patients with technology dependence. In this 4-week clinical rotation, the opportunities for tracheostomy management depended on the number of patients with technology dependence, which varied every month. Having already gained some fundamental knowledge and skills from the flipped classroom, residents were better equipped to practice routine tracheostomy care or participate in the tracheostomy emergencies when the opportunities arose during the rotation. We offered further teaching in the form of simulation-based instruction during the last week of the rotation. Residents underwent simulation-based assessment during this face-to-face teaching, received immediate constructive feedback during debriefing following simulation, and completed a curriculum evaluation along with the post-SE assessment.

## Results

All 107 residents from baseline and intervention groups completed the SE survey. SE scores increased at postrotation across all four domains of SE. Residents receiving simulation reported greater improvement of overall SE scores compared to the baseline group, with a median difference of 0.4 (*p* < .0001; Table). Residents had an overall knowledge assessment score of 75%. All 40 residents who completed the module had overall scores greater than 80% during the simulation assessment. The simulation performance scores were high, crossing the mastery threshold (80%) in the routine change of tracheostomy. The routine tracheostomy care scenario encompassed residents’ ability to demonstrate steps with routine tracheostomy change in addition to appropriate depth of suctioning, with overall scores of 87%. Residents had to define the steps of emergency tracheostomy, obstructed tracheostomy (88%), and CPR (81%). The tracheostomy emergency simulation tested each resident's ability to define and demonstrate specific skills pertaining to the four steps to follow in a tracheostomy emergency. The application of these skills in executing the cognitive, technical, and behavioral skills required for managing a tracheostomy emergency was also assessed. Residents rated the quality of the curriculum (1 = *least useful,* 5 = *greatly useful*), with 4.8 for content, 4.4 for learning environment, and 4.0 for overall quality. Narrative comments from the feedback survey showed residents highly appreciated this teaching. Some of their comments are categorized below by specific topic:

• Timing of simulation:
  ○ “Early in the rotation would be ideal.”
  ○ “It would be nice to have something like this earlier in the rotation perhaps 2nd week because it's really useful.”
• Desire for more practice:
  ○ “Perhaps would be beneficial to have this twice during TICU.”
  ○ “It would help to have more protected time to do simulations more frequently during the block.”
  ○ “Doing some of these things on a real patient.”
• Educational content:
  ○ “15 min video was awesome, maybe including other videos about common trach outcomes especially wounds around neck brace etc., as a way to keep in mind trach morbidity.”
  ○ “Good level of teaching for my current knowledge level.”
  ○ “Excellent use of trach change in emergency scenario to facilitate learning.”
• Supportive learning environment:
  ○ “The group and 1 on 1 training are fantastic and I think it is essential.”
  ○ “Amazing simulation, very supportive instructors.”
• Feedback is critical to learning:
  ○ “I felt the feedback on sim was very helpful and empowered me to better manage patient emergencies.”
  ○ “Greatly appreciate all the sim training this rotation, feel confident for my next rotation.”
  ○ “Wonderful instructors who are very good at giving feedback.”

**Table.**
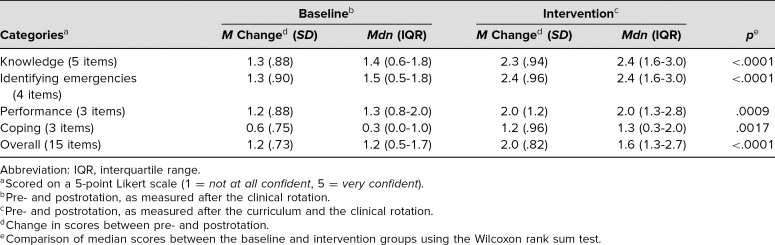
Comparison of Average Change in Self-Efficacy Scores Between Baseline and Intervention Groups

## Discussion

To address previous concerns about the need for improved airway management skills in pediatric care providers,^21–23^ as highlighted by the Accreditation Council for Graduate Medical Education,^23,24^ we designed our curriculum specifically to improve skills and confidence of residents working in ICU units and caring for patients with medical complexity. We gave thorough consideration to developing the components of our curriculum to encompass routine care of tracheostomy and emergency management of tracheostomy. Our methods aligned with what was previously known about simulation providing a reassuring positive environment that allows for acquisition of skills and promotes SE and competence with management of tracheostomies.^14^

Tracheostomy emergencies, although catastrophic, are rare occurrences. Although numerous efforts have been made to address this issue,^25,26^ primary care providers often feel overwhelmed managing children with complexities^27^ and need a simplified approach. By simplifying the steps required for the complex task of tracheostomy care while promoting SE, our intervention directly addressed this issue in developing hands-on skill. Working with their peers in the group learning activity of routine tracheostomy care was extremely useful for familiarizing residents with equipment, in addition to developing SE and addressing anxiety and fears associated with managing these patients. However, the one-on-one simulation of managing a tracheostomy emergency was most useful in application of cognitive skills, rendering the simulation scenario realistic to actual patient encounters and improving motivation for learning new skills.

By featuring an actual patient receiving routine tracheostomy care in the video, our approach allowed for visual demonstration of skills along with the ability for asynchronous learning. As most of our learners were first-year residents, we had to ensure that they were not overwhelmed. We did so by providing a nurturing and supportive environment during simulation, with constructive feedback to improve future performance. Hands-on training with debriefing facilitated many opportunities to learn and improve resuscitation skills, although this benefit was not a primary objective of our study. All of our learners enjoyed the learning experience, with some requesting more practice. However, the constraints on time in a busy ICU setting did not permit multiple or repeat simulation testing within the rotation. As part of future directions, we will be implementing this intervention at another hospital site to test for generalizability of our approach. In addition, we will perform video scoring of simulation performance to objectively assess performance of tasks during simulation.

### Limitations

Our study did not assess sustainability of skills developed as a result of the curriculum, as our assessments were performed immediately after the simulation, and the improvements demonstrated may have been partially attributed to patient exposure during rotation. Seeking SE feedback right after debriefing from simulation might induce artificially high levels of confidence. Social desirability can also affect self-reported survey results. Performance in simulation does not always reflect how learners will perform in the clinical domain. Due to the nature of our clinical rotation, our participants were limited to first-year residents. This curriculum highlights the basic skills for managing tracheostomy; it is by no means comprehensive and does not cover unique aspects of caring for an older child or adult with a tracheostomy.

## Appendices

Self-Efficacy Survey.docxVideo Module.mp4Knowledge Assessment.docxSimulation Instruction.docxSimulation Assessment.docxCurriculum Feedback Survey.docx All appendices are peer reviewed as integral parts of the Original Publication.
