# Supplementary material for: Improving Resident Self-Efficacy in Tracheostomy Management Using a Novel Curriculum
Source: MedEdPORTAL. 2020 Nov 3;16:11010. doi: 10.15766/mep_2374-8265.11010 (PMC7666842; doi:10.15766/mep_2374-8265.11010)
Supplement: Supplementary file 1 — Self-Efficacy Survey.docxVideo Module.mp4Knowledge Assessment.docxSimulation Instruction.docxSimulation Assessment.docxCurriculum Feedback Survey.docx [file mep_2374-8265.11010-s001.zip › A. Self-Efficacy Survey.docx]

**Resident Self- Efficacy Survey with Tracheostomy Care – Appendix A**

Rate you degree of confidence by recording a number from 1-5 using the scale given below

1 2 3 4 5

Not at all confident Fairly confident Very confident

| **Knowledge** | **1** | **2** | **3** | **4** | **5** |
| --- | --- | --- | --- | --- | --- |
| When asked about my patient’s tracheostomy, I usually can provide the accurate information regarding the following, I | | | | | |
| know the type and size of tracheostomy cuffed or un-cuffed |  |  |  |  |  |
| know how often the tracheostomy tube is changed |  |  |  |  |  |
| know how to change the tracheostomy tube |  |  |  |  |  |
| know how to provide routine care of the tracheostomy site |  |  |  |  |  |
| know the indications for tracheostomy for the patient |  |  |  |  |  |
| **Identifying emergencies** | | | | | |
| If your patient had a problem with their trach and needed immediate help can you performing the following: | | | | | |
| know the type and size of tracheostomy cuffed or un-cuffed |  |  |  |  |  |
| know how often the tracheostomy tube is changed |  |  |  |  |  |
| know how to change the tracheostomy tube |  |  |  |  |  |
| know how to provide routine care of the tracheostomy site |  |  |  |  |  |
| know the indications for tracheostomy for the patient |  |  |  |  |  |
| **Identifying emergencies** | | | | | |
| When asked about my patient’s tracheostomy, I usually can provide the accurate information regarding the following, I | | | | | |
| Identify airway compromise in a timely fashion |  |  |  |  |  |
| know when the tracheostomy is dislodged out of the airway |  |  |  |  |  |
| Know how to clear a blocked tracheostomy |  |  |  |  |  |
| **Performance** | | | | | |
| If your patient had a problem with their trach, can you perform the following | | | | | |
| Replace the tracheostomy tube that has de-cannulated |  |  |  |  |  |
| Seek appropriate help |  |  |  |  |  |
| Secure ties around the tracheostomy |  |  |  |  |  |
| know how to provide routine care of the tracheostomy site |  |  |  |  |  |
| know the indications for tracheostomy for the patient |  |  |  |  |  |
| **Coping** | | | | | |
| When dealing with an airway emergency on my patient | | | | | |
| I can stay calm when I see my patient with an airway emergency |  |  |  |  |  |
| I can successfully administer CPR |  |  |  |  |  |
| I can successfully manage my anxiety and manage the airway |  |  |  |  |  |
| **PGY: Specialty**  **How many patients with tracheostomy have you managed in the past?** | | | | | |
