# Supplementary material for: Improving Resident Self-Efficacy in Tracheostomy Management Using a Novel Curriculum
Source: MedEdPORTAL. 2020 Nov 3;16:11010. doi: 10.15766/mep_2374-8265.11010 (PMC7666842; doi:10.15766/mep_2374-8265.11010)
Supplement: Supplementary file 1 — Self-Efficacy Survey.docxVideo Module.mp4Knowledge Assessment.docxSimulation Instruction.docxSimulation Assessment.docxCurriculum Feedback Survey.docx [file mep_2374-8265.11010-s001.zip › C. Knowledge Assessment.docx]

**Knowledge assessment for Tracheostomy education – Appendix C**

1) The following are **not** indications for tracheostomy

A Managing secretions in a patient with neuro-muscular weakness

B Managing secretions in a patient with neuro-muscular weakness

C Preventing aspiration in a patient with swallow dysfunction

**D Providing a stable airway in a patient with acute retropharyngeal abscess**

2) Following a tracheostomy surgery the first tracheostomy change should be done by

1. At home by community nursing team 1-2 weeks following surgery
2. **ENT specialist in OP clinic 1-2 weeks after surgery**
3. Parents who have been trained within 1 month of surgery
4. PCP in the Complex care/neuro-disability clinic in < 1 month after surgery

3) Tracheostomy tube in an otherwise stable patient should be changed out **(Select all that apply)**

**A** By home health nurses every 2 weeks

B **By parents who are tracheostomy trained every month**

C Community nursing that visit home every 2 weeks

D PCP in their office every month

4) The following statements are **TRUE** about routine tracheostomy change

**A Cuffed tubes should be inflated with sterile water prior to insertion to check if cuff patent**

B Tracheostomy tube should be changed out every 2 months

C When replacing a tracheostomy tube with a one person technique you might have to use both hands to remove the ties as the neck can be short in infants

D Suctioning should be vigorous to the estimated depth to remove secretions

5) The following is the commonly followed sequence to follow in a tracheostomy emergency

**A Suction Trach, Bag through Trach, Change out Trach, start CPR, call code team if needed**

B Suction, Change out Trach, Bag through Trach, start CPR, call CODE if needed

C Suction, Change out Tube, start CPR, Call code if needed

D Suction, Change out Trach, Bag through Trach, call code team

6a) You have been called to the beside of a 2 yr. old with tracheostomy and ventilator dependence, who was admitted with RSV infection. His ventilator has been alarming with desaturations down to 80’s for the last 15 minutes. His vitals are Sp02 82%, RR at 60/ min, HR 157/ min, BP 80/64 with substantial work of breathing. His nurses have disconnected the vent and report – difficulty with suctioning and resistance with bagging through the tracheostomy. Which of the following is the **CORRECT** diagnosis

A Air leak from tracheostomy cuff

B Dislodged tracheostomy

**C Obstructed tracheostomy**

D Smaller size tracheostomy that is inappropriate

6b) You attempt suctioning through the tracheostomy and get minimal secretions, and perform bagging with no improvement with saturations the next **BEST s**tep would be to

A Administer albuterol nebulizer along with saline back to back and get the respiratory therapist to re-adjust tracheostomy

B Administer CPR and call the CODE team

C Increase the pressure settings on the ventilator and obtaining a blood gas

D Reconnect the ventilator to see if the patient improves with normalizing his vent settings

**E Replace with a new tracheostomy tube and ensure improvement of saturations prior to reconnecting the ventilator**

7 a) Zoe is an 18 mo, previous 23 weeker with severe BPD and pulmonary hypertension, Trach (3.0 size bivonna cuffed) with ventilator dependence, and gastrostomy dependence with developmental delay, admitted for the last 2 months in respiratory failure from H.influenza. Recently she developed narcotic habituation for which she is on a gradual wean of sedation. She has progressively needed more O2 since the morning. You are the night resident that has been called to assess her. The next **BEST** step in her management is

**A. Assess for airway by checking air entry, work of breathing, cyanosis and position of trach**

B. Prepare to change out tracheostomy and have a smaller size tracheostomy ready

C. Suction her tracheostomy, and bag through her tracheostomy if suction does not help

D. Treat with a prn dose of methadone for agitation and re-assess in 30 mins.

7 b) As you obtain further history the nurse states that his pressure settings on the vent has been alarming with low minute ventilation and she suspects an air leak. The following is the **MOST** probable cause for his change in status

1. Aggressive sedation wean

**B. Dislodgement of tracheostomy**

C. Low cuff pressure in the tracheostomy tube

D. Water in the vent tubing

8) 24 mo. with Pierre Robin syndrome comes to your clinic for follow up. He has a tracheostomy (4.0 Pedi Bivona uncuffed) with ventilator dependence at night and a humidified trach collar during the day. His family had to travel for the last hour to get to your clinic. He has very active lately and been pulling on his G-tube and his tracheostomy. Just before you enter the room, he accidentally pulled out his tracheostomy. His mother tried to re-insert the same size tube and is unable to do so. The next **BEST** step would be to

1. Initiate a code.
2. **Insert a 3.5 size Pedi Bivonna**
3. Ensure tracheostomy cuff is working before insertion
4. Intubate the patient to secure airway

9) You are the ED resident, called to attend a 6 mo with HLH s/p palliation with Glen anastomosis, with tracheostomy (3.0 Pedi Bivona uncuffed), with OSA, vent dependence and gastrostomy dependence was brought in by parents as they are concerned about him. He was brought into ED with high fever x 2 days, which was initially thought to be viral. He was tolerating feeds till last night, but has been fussy since this morning. On arrival his saturations are 72 (usually high low 80’s), with a HR of 60 beats/ min, CRT of 4 secs and a weak femoral pulse. The **NEXT** best step in management is

1. Administer early ceftriaxone at 100mg/kg/dose IV ceftriaxone
2. Administer DC shock at 2 J/kg
3. **Initiate CPR at the rate of 15:2 compressions at a rate of 100 compressions/ min**
4. Give a fluid bolus of 10 mls/ kg of normal saline and repeat again in 2 mins
