# Supplementary material for: Improving Resident Self-Efficacy in Tracheostomy Management Using a Novel Curriculum
Source: MedEdPORTAL. 2020 Nov 3;16:11010. doi: 10.15766/mep_2374-8265.11010 (PMC7666842; doi:10.15766/mep_2374-8265.11010)
Supplement: Supplementary file 1 — Self-Efficacy Survey.docxVideo Module.mp4Knowledge Assessment.docxSimulation Instruction.docxSimulation Assessment.docxCurriculum Feedback Survey.docx [file mep_2374-8265.11010-s001.zip › D. Simulation Instruction.docx]

| **Appendix D: MedEdPORTAL Simulation Case Template**  **SIMULATION CASE TITLE: Obstructed Tracheostomy**  **AUTHORS: J Benjamin, K. Roy, S Kumar, G. Paul MD, E Charles, E Miller, H, Narsi- Prasla, J. D. Mahan MD, S. Thammasitboon** | |
| --- | --- |
| **Patient Name: John**  **PATIENT AGE: 2yr**  **CHIEF COMPLAINT: respiratory distress after procedure requiring sedation and returning from MRI suite** | |
|  | |
| **Brief narrative description of case** | 2 yo previous 29 week premie with, grade 4 IVH, CLD,  Pulmonary hypertension on CPAP, s/p tracheostomy for multiple previous failed extubation attempts. He underwent recent sedation for a brain MRI and has increased secretions for the last 3 days. Over the course of the night he has needed more O2 and his pressures were increased after a recent gas showed respiratory acidosis. The nurse pages you that his saturations have been dropping below 90% |
| **Primary Learning Objectives** | **Cognitive:**   - Learners will perform an initial airway assessment - Learners will recognize signs of respiratory distress (no chest rise, baby blue) - Learners will recognize mucous plug and indicate need to suction and follow steps with trach emergency. - Learners will recognize cause for no improvement and notice resistance with bagging - Learners will recognize pulselessness - Learners will verbalize the steps to follow in a patient with pulselessness – initiate code, CPR   **Technical:**   - Residents will suction to correct depth and attempt bagging through tracheostomy - Residents will provide appropriate breaths via ambu bag and note no chest rise. - Residents will change out tracheostomy tube and assist in 2- person technique   **Behavioral:**   - Residents will work together as a team - Residents will communicate with each other effectively during emergency - Residents will have the ability to perform during the emergency with effective crises management skills – Clear communication, closed loop communication |
| **Critical Actions** | - Identify signs and symptoms of respiratory distress - Follow steps of tracheostomy emergency - Assess signs of respiratory distress - Recognize deterioration in clinical signs changes out tracheostomy tube - Recognizes bradycardia as worsening of |
| **Learner Preparation** | 2 yo previous 29 week premie with, grade 4 IVH, CLD,  Pulmonary hypertension on CPAP, s/p tracheostomy for multiple previous failed extubation attempts. He underwent recent sedation for a brain MRI and has increased secretions for the last 3 days. Over the course of the night he has needed more O2 and his pressures were increased after a recent gas showed respiratory acidosis. The nurse pages you that his saturations have been dropping below 90%   - Remind residents to **think and speak out loud what they are doing and thinking** |

| **Initial Presentation** | | | |
| --- | --- | --- | --- |
| **Initial vital signs** | The vital signs, fetal heart rate tracing and pattern below will be presented on the monitor.  **HR 160/ min, Sats 72/ min, RR 70/min** | | |
| **Overall Appearance** | Baby with tracheostomy lying in bed, cyanosis around mouth. | | |
| **Actors and roles in the room at case start** | Prior to the start of the simulation, the facilitator will present the patient history to the learner and encourage the learner to think out loud and speak through their responses to help facilitators to guide the learner. A facilitator who is a bed side nurse will be available to assist, but will only preform actions as directed by the learner.  Learners will perform an initial assessment and comment on the saturations being low and start performing suctioning to appropriate depth. As suctioning does not produce the desired improvement, residents will then move onto bagging. With no outcome of improving saturations, the nurse helper will offer to carry on bagging as the learner prepares the tracheostomy for a tracheostomy change.  With the tracheostomy change the saturations will improve, but the monitor will show bradycardia for learners to recognize decompensation as a result of decompensation. | | |
| **HPI** | Facilitator will give the following information:  *You are being called to the bedside of this patient in TICU*  2 yo previous 29 week premie with, grade 4 IVH, CLD,  Pulmonary hypertension on CPAP, s/p tracheostomy for multiple previous failed extubation attempts. He underwent recent sedation for a brain MRI and has increased secretions for the last 3 days. Over the course of the night he has needed more O2 and his pressures were increased after a recent gas showed respiratory acidosis. The nurse pages you that his saturations have been dropping below 90%    Learners will then perform a physical examination which must include chest, lungs for breath sounds and cyanosis around lips. | | |
| **Past Medical/Family History** | **Past Surgical History** | **Medications** | **Allergies** |
| Previous 29 weeker | Tracheostomy, recent sedation with MRI and increased respiratory distress following sedation. | None | None |
| **Physical Examination** | | | |
| **General** | Cyanosis, respiratory distress | | |
| **HEENT** |  | | |
| **Neck** |  | | |
| **Lungs** | Reduced air entry, cyanosis | | |
| **Cardiovascular** | S1, S2 normal, Regular rate and rhythm, no murmurs/rubs/gallops | | |
| **Abdomen** | Normal | | |
| **Neurological** | Alert but in respiratory distress | | |
| **Skin** |  | | |
|  | | | |

| **Instructor Notes - Changes and CASE Branch Points**  *This section should be a list with detailed description of each step than may happen during the case. If medications are given, what is the response? Do changes occur at certain time points? Should the nurse or other participant prompt the learners at given points? Should new actors or participants enter, and when? Are there specific things the patient will say or do at given times? There are a few examples given, but it is expected that most cases will have many more changes and potential branch points..* | | |
| --- | --- | --- |
| **Intervention / Time point** | **Change in Case** | **Additional Information** |
| Learners are told that saturations have dropped  Baseline vital signs:  **HR 160/ min, Sats 90% , RR 70/min, HR 120** | Learners must:   - Perform initial assessment of airway and breathing - Comment on no improvement of saturations - List the steps to follow in tracheostomy emergency - Perform suctioning to appropriate depth - Attempt bagging as saturations do not improve | Identify higher pressures on CPAP machine  Identify higher pressure for bagging when bagging through tracheostomy |
| Learners are told that saturations are not improving and that the patient is continuing to desaturate  Vital signs:  **HR 160/ min, Sats 72% , RR 80/min, HR 140** | Learners must:   - List the next step in management of Trach emergency – which is need for change of tracheostomy - Give clear instruction to the nurse to continue bagging | Nurse will offer to bag when learner prepares tracheostomy |
| Saturations improve as tracheostomy is changed  Vital signs: **HR 160/ min, Sats 94% , RR 80/min, HR 42/ min** | Learners must:   - Recognize bradycardia - Worsening of status due to decompensation - Continue bagging or assigning nurse helper to bag , when they feel for a pulse - Call for CODE Obtain pulse oximetry - Indicate need for CPR | If learners do not recognize bradycardia, nurse will cue learner to the patient being less active and being more sleepy  If learner did not ask for bagging, nurse will clarify if the patient needs to continue bagging |
| As resident recognizes bradycardia, calls for CODE and starts CPR with chest compressions and bagging, scenario will end | Learner must:   - Determine need for CPR |  |

**Ideal Scenario Flow**

Learners will identify signs of respiratory distress and recognize airway related emergency and follow through with steps in managing a tracheostomy emergency and identify blocked tracheostomy.

**Anticipated Management Mistakes**

1. Failure to identify respiratory distress and not addressing tracheostomy emergency and advancing through steps to follow in an emergency.
2. Failure to take patient off ventilator, i.e CPAP( instead of bagging to hyperventilate a patient who is hypoxic )
3. Failure to correctly identify bradycardia as a sign of decompensation of a tracheostomy emergency scenario. At the start of simulation, learners get cued into the desaturations and may only be monitoring this parameter instead of scanning the whole monitor for abnormal vitals in case of a decompensation
4. Failure to call for CODE swiftly when they notice bradycardia, or not remembering to check pulse before initiation of CPR
5. Mistaking decompensation as a result of tracheostomy change and being distracted about airway being the problem, when the issue is with circulation
