# Supplementary material for: Improving Resident Self-Efficacy in Tracheostomy Management Using a Novel Curriculum
Source: MedEdPORTAL. 2020 Nov 3;16:11010. doi: 10.15766/mep_2374-8265.11010 (PMC7666842; doi:10.15766/mep_2374-8265.11010)
Supplement: Supplementary file 1 — Self-Efficacy Survey.docxVideo Module.mp4Knowledge Assessment.docxSimulation Instruction.docxSimulation Assessment.docxCurriculum Feedback Survey.docx [file mep_2374-8265.11010-s001.zip › E. Simulation Assessment.docx]

**Resident Trach Education Check list following sim education – Appendix E**

**Routine care – Instruct residents to demonstrate routine change of tracheostomy after teaching scenario**

Scoring 0 = not done, 1= done correctly but out of order, 2= done correctly in order N = Not applicable

| **Scenario 1** | **Routine care** | Score | Comments |
| --- | --- | --- | --- |
| **(Cognitive skills)** | Knows size Type of tracheostomy |  |  |
|  | Cuffed or un-cuffed |  |  |
| **(Technical skills)** | Can get required supplies for trach change including smaller size trach  Trach tube including smaller size, trach ties, suction catheter, lubrication, sterile water for cuffed trach, lubrication, Syringes, neck roll |  |  |
|  | Places neck roll under patient |  |  |
|  | Can assist in 2 person technique for trach change |  |  |
|  | Holds tube in place after insertion |  |  |
|  | Removes obturator after insertion |  |  |
|  | Secure ties |  |  |

| **(Behavioral skills** | Score |  |
| --- | --- | --- |
|  | Familiarity with environment |  |
| Unfamiliar with equipment needed for Trach change | 1 2 3 4 5 NA | Familiar with equipment |
|  | Team player |  |
| Cannot assist in 2 person technique | 1 2 3 4 5 NA | Can assist in 2 person technique |
| Unfamiliar with securing ties | 1 2 3 4 5 NA | Can demonstrate how to secure ties |

**Scenario 2 Obstructed Trach leading onto CPR**

| **Scenario 2** | **Obstructed trach** | Scoring |
| --- | --- | --- |
| Cognitive skills | Identifies Airway compromise |  |
|  | Suctions to correct depth |  |
|  | Verbalizes the need for replacement trach |  |
|  | Verbalizes 4 steps to follow in emergency |  |
| Technical skills | Assess breathing, feels for pulse |  |
|  | Verbalize HR on monitor |  |
|  | Provides suction to correct depth |  |
|  | Bag through Trach |  |
|  | Replaces tracheostomy |  |
|  | Hold tube in place after insertion |  |
|  | Removes obturator immediately after insertion |  |
|  | Secures ties |  |

Scoring 0 = not done, 1= done correctly but out of order, 2= done correctly in order N = Not applicable

| **Familiar with the environment** | | |
| --- | --- | --- |
| Unfamiliar with equipment, cannot find bag and trach | 1 2 3 4 5 | Familiar with suctioning, trach  Able to find all emergency supplies in a timely fashion (bag, trach). |
| **Anticipates and plans ahead for crises** | | |
| Slow to recognize need for to change tracheostomy (> 5 min ) | 1 2 3 4 5 | Quickly identifies problem and replaces trach, does not fixate on other distractors- troubleshooting ventilator, suctioning, etc. |
| **Communicate effectively, if back up caregiver present** | | |
| Mumbles, speaks quietly, unable to explain situation and interventions to backup caregiver | 1 2 3 4 5 NA | Communicates (verbalizes) all actions to backup caregiver before doing, explains situation and interventions clearly |
| **Assume a leadership role** | | |
| Seems unable to take control of the situation, does not delegate roles and tasks for additional help | 1 2 3 4 5 | Takes charge of the crisis- takes appropriate steps to manage the situation, delegates roles and tasks for additional help |
| **Utilizes available resources** | | |
| Doesn’t elicit help from team  Doesn’t utilize emergency equipment  (bag mask, opened) | 1 2 3 4 5 | Utilizes available resources ( help from team), utilizes emergency equipment ( bag mass,xopenex) |
| **Calls for help early enough if needed** | | |
| Doesn’t call for help and/or 911 if unable to reinsert tube | 1 2 3 4 5 NA | Calls for help if unable to troubleshoot and change tube in 5 min |
| **Maintains calm behavior** | | |
| Behaves inappropriately , extremely distressed /agitated | 1 2 3 4 5 | Remains calm during crisis, ability to perform during the emergency |

| **Testing scenario** | **CPR** | Score | Comments |
| --- | --- | --- | --- |
| **Cognitive skills** | Recognizes signs of apnea, cyanosis, pulselessness |  |  |
|  | Recognizes and verbalizes need for CPR |  |  |
|  | Calls for help |  |  |
| **Technical skills** | Assesses for breathing, pulse, sats and HR |  |  |
|  | Administers CPR 100 Compressions/ min |  |  |
|  | Provides appropriate breath via ambu bag to trach or mouth with stoma occlusion |  |  |

**CPR**

| **Familiar with the environment** | | | |
| --- | --- | --- | --- |
| Unfamiliar with CPR | 1 2 3 4 5 | Familiar with administering CPR | |
| **Anticipates and plans ahead for crises** | | | |
| Slow to recognize need for help when not improving | 1 2 3 4 5 | When no improvement after CPR initiated, calls 911 for help early | |
| **Communicate effectively** | | | |
| Mumbles, speaks quietly, unable to explain situation and interventions to backup caregiver | 1 2 3 4 5 NA | Communicates (verbalizes) all actions to backup caregiver before doing, explains situation and interventions | |
| Distribute workload optimally | | | |
| Does not assign tasks to team members gets distracted with unimportant tasks. Multitasks when not necessary | 1 2 3 4 5 NA | Assigns appropriate tasks to back up caregiver ( finding supplies, calling EMS), no multitasking | |
| **Assume a leadership role** | | | |
| Seems unable to take control of the situation – multitasks, unable to assign roles and delegates tasks to helper | 1 2 3 4 5 | Takes charge of the crisis -does not multitask, assigns roles and delegates tasks to helper | |
| **Allocates attention wisely** | | | |
| Gets distracted easily and fails to initiate CPR in a timely manner. Becomes fixated on airway issues with delay in CPR | 1 2 3 4 5 | Allocates attention wisely, focuses on providing CPR. Does not delay initiation of CPR | |
| **Utilizes available resources** | | | |
| Doesn’t elicit help from co- caregiver  Doesn’t utilize emergency equipment ( bag mass, ) | 1 2 3 4 5 | Utilizes available resources ( help from caregiver), utilizes emergency equipment ( bag mass,) | |
| **Calls for help early enough if needed** | | | |
| Doesn’t call for help | 1 2 3 4 5 NA | | Calls for help within 2-3 minutes |
| **Maintains calm behavior** | | | |
| Behaves inappropriately , extremely distressed /agitated | 1 2 3 4 5 | | Remains calm during crisis, ability to perform during the emergency |
