# Supplementary material for: Improving Resident Self-Efficacy in Tracheostomy Management Using a Novel Curriculum
Source: MedEdPORTAL. 2020 Nov 3;16:11010. doi: 10.15766/mep_2374-8265.11010 (PMC7666842; doi:10.15766/mep_2374-8265.11010)
Supplement: Supplementary file 1 — Self-Efficacy Survey.docxVideo Module.mp4Knowledge Assessment.docxSimulation Instruction.docxSimulation Assessment.docxCurriculum Feedback Survey.docx [file mep_2374-8265.11010-s001.zip › F. Curriculum Feedback Survey.docx]

**Evaluation of Tracheostomy Curriculum – Appendix F**

Rate the tracheostomy education on a scale of 1-5

**Process (1 = little, 5= Greatly)**

1. How much does observing other providers perform tasks during tracheostomy emergencies facilitate your learning?

1 2 3 4 5

1. How much does the constructive feedback during simulation facilitate your learning?

1 2 3 4 5

1. How much does the successful completion of tasks in the simulation scenario enable success during the testing scenario?

1 2 3 4 5

**Learning environment (1 = Extremely Nervous 5 Well supported)**

1. Please describe your feeling while performing tasks during simulation

1 2 3 4 5

1. Please describe your feeling during de-brief following simulation

1 2 3 4 5

**Outcome (1 = Disagree 5= Strongly agree)**

1. I feel confident in applying knowledge and skills learned to patient care

1 2 3 4 5

1. I feel empowered to manage a patient in tracheostomy emergencies

1 2 3 4 5

**Content (1 = Disagree 5= Strongly agree)**

1. The video instruction was clear with demonstration of techniques required for tracheostomy management

1 2 3 4 5

1. The curriculum provides useful knowledge content

1 2 3 4 5

1. This curriculum was worth my time commitment

1 2 3 4 5

Please give us any comments on how we can improve this curriculum

--------------------------------------------------------------------------------------------------------------------------------------------------------------------------------------------------------------------------------------------------------------------------------------------------------------------------------------------------------------------------------------------------------------------------------
